# Piloting the Impact of Three Interventions on Guaiac Faecal Occult Blood Test Uptake within the NHS Bowel Cancer Screening Programme

**DOI:** 10.1155/2015/928251

**Published:** 2015-10-07

**Authors:** Becky White, Emily Power, Monika Ciurej, Siu Hing Lo, Katherine Nash, Nick Ormiston-Smith

**Affiliations:** ^1^Cancer Research UK, Angel Building, 407 St. John Street, London EC1V 4AD, UK; ^2^Health Behaviour Research Centre, University College London, Gower Street, London WC1E 6BT, UK

## Abstract

This study evaluated the impact of three interventions on uptake of the guaiac faecal occult blood test (gFOBT) in Greater London. The interventions were designed to improve awareness and understanding of the NHS Bowel Cancer Screening Programme (BCSP) and assist stool sampling. Logistic regression analysis of BCSP London data (*N* = 205,541 invitees aged 60–74) compared uptake at 12 weeks between intervention groups and a control group, sent kits as usual between January-April 2013 and January-April 2014. An endorsement flyer, included with gFOBT kits, had no impact on uptake (*P* = 0.68). In 60–69-year-olds, there was a small but significant increase in modelled uptake amongst invitees sent both the flyer and a kit enhancement pack compared with controls (45.1% versus 43.4%, OR = 1.07, *P* = 0.047). In North East London, the flyer together with outdoor advertising was associated with a small but significant increase (45.6% versus 43.4%, OR = 1.09, *P* = 0.027). The largest increases were seen when all three interventions (flyer, pack, and advertising) were combined (49.5% versus 43.4%, OR = 1.28, *P* < 0.001). The increased uptake in the intervention groups was largest in “first-timers” and smaller amongst previous nonresponders and previously screened invitees.

## 1. Introduction

The NHS Bowel Cancer Screening Programme (BCSP) invites eligible adults aged 60–74 in England to complete a guaiac-based faecal occult blood test (gFOBT) every two years. The English programme was launched in 2006 for 60–69-year-olds and has since been extended to 70–74-year-olds. Initial randomised controlled trials found a reduction in mortality risk from bowel cancer using gFOBT screening [1–4], with a systematic review of these trials finding a 15% relative risk reduction in bowel cancer mortality in studies using biennial screening [5]. It has been predicted that the biennial gFOBT bowel cancer screening programme could save 1,800 to 2,400 lives each year by 2025 in England [6].

Overall, gFOBT screening uptake in the NHS BCSP is around 54% [7], which is lower than the English NHS cervical screening and breast cancer screening programmes [8, 9]. Uptake is lower amongst more socially deprived invitees, unmarried invitees, and males and in more ethnically diverse areas [7, 10–12]. Uptake is much higher amongst invitees who have been adequately screened before [13]. Overall, research suggests that uptake increases with age, although there are discrepancies, with some studies reporting no association [10] and others reporting lower uptake amongst younger invitees [12], higher uptake with age in males [7], or a peak in uptake at 64–66 years [13].

Relatively few interventions designed to improve uptake of gFOBT screening have been conducted in the UK [14], although there has been some success using endorsement letters and additional information leaflets [15–17]. Hewitson et al. found that a GP endorsement letter and an enhanced screening information leaflet (providing further detail about how to complete the kit) each increased uptake of gFOBT by approximately 6 percentage points and had an additive impact on uptake when trialled together [15].

Commonly reported barriers centre on the procedure required to complete the test, for example, distaste and embarrassment around sampling and storing faecal samples, concerns about completing the test at home rather than in a formal health setting, and misunderstanding the instructions [14, 18–21]. Published research into the effectiveness of providing practical aids in the stool sampling process is limited. A faeces collection paper aiming to address discomfort with the sampling process has been tested in the Netherlands [22]. It was not associated with significantly increased participation in Faecal Immunochemical Test (FIT) bowel cancer screening, although the authors provided little information about the collection paper tested and did not collect any data on reported use.

Public awareness and understanding of the test are likely to be an issue for some, given the programme's relative infancy compared to those for breast and cervical cancer and given that it is the first national screening programme for men in the UK. To our knowledge, no published studies have yet assessed the impact of a bowel screening advertising campaign on uptake in the UK. Nevertheless, the positive effects that mass media campaigns can have on health behaviours have been reported previously, including short term increases in breast and cervical cancer screening uptake in countries with organised screening services [23]. A previous study found that media coverage of the UK Flexible Sigmoidoscopy Trial was associated with a small but positive increase in early uptake of gFOBT screening, particularly among previous nonresponders [24].

This study reports the findings from a bowel cancer screening service improvement pilot that ran from January to April 2014. The project aimed to increase uptake of the NHS gFOBT in Greater London, by raising awareness of the programme and reducing key barriers to completion. The project trialled different combinations of three interventions, which attempted to increase awareness and understanding about screening, build on the previous success of GP endorsement, and address practical issues people may have with the test.

## 2. Materials and Methods

### 2.1. Setting

The pilot was delivered by Cancer Research UK (CRUK), who worked with NHS England (London region), Public Health England, the Department of Health, and the English NHS Bowel Cancer Screening Programme (BCSP). It targeted men and women aged 60–74 years in Greater London, who were due to receive their kit from the BCSP during the study period.

The BCSP sends all eligible men and women an invitation to participate in bowel screening, which also includes an information leaflet about bowel cancer and the screening process. It then sends a second letter one to two weeks later, containing the cardboard gFOBT kit, cardboard sticks, instructions for completing the kits, and a prepaid return envelope. Invitees are instructed to collect two small faecal samples each from three separate bowel motions, to spread onto six different windows on the kit.

Ethical approval was not required for this study, as it was an evaluation of a service improvement pilot.

### 2.2. Design

Different combinations of the following three interventions were trialled: a CRUK endorsement flyer, “kit enhancement packs,” and an outdoor advertising campaign. These interventions are described in more detail below.

Table 1 illustrates how different combinations of the interventions were piloted in different intervention groups. Intervention groups A and B were trialled across all of Greater London (including North East London), whilst groups C and D were tested in North East London only, alongside the advertising campaign that was simultaneously conducted in North East London. Differences in uptake between intervention groups A and B and C and D could partly be due to particular characteristics of the North East London population that are known to affect bowel screening uptake. These characteristics include ethnicity and/or marital status [10, 11], which we were unable to account for in the logistic regression analysis, and therefore any comparisons of uptake between different intervention groups must be made cautiously.

### 2.3. Interventions

#### 2.3.1. CRUK Endorsement Flyer

An A5 flyer (Supplementary Figure 1 in Supplementary Material available online at  http://dx.doi.org/10.1155/2015/928251) was included with the routine gFOBT kit mailings from the London BCSP Hub, which administers the bowel cancer screening programme regionally. The flyer was designed to increase understanding and encourage people to consider completing the test by emphasising its effectiveness, privacy, and ease of use and providing information about how many other people complete screening in London. The flyer also provided an endorsement of screening by CRUK and a reminder that participation in the programme was the recipient's own choice. Printing costs for this intervention were minimal, and there was no extra postage cost as they were included in the routine gFOBT kit mailings.

#### 2.3.2. “Kit Enhancement Packs”

Packs (Supplementary Figure 2) included latex-free gloves and “poo catchers,” which slip over the toilet seat and are designed to make sample collection easier. Three sets of each were included in each pack, one for each stool sample required for the gFOBT screening kit, and “poo catchers” came with simple, visual instructions. The packs were distributed through the London BCSP Hub two days after the gFOBT kits had been mailed. Feedback about the packs from focus group testing commissioned by CRUK indicated that people would value both the gloves and “poo catchers.” Plus, participants who tested the “poo catcher” said it was easy to use, with one saying it was “*much better than hunting around for some sort of vessel.*” The production costs were £1.53 per pack (including gloves and “poo catcher”).

#### 2.3.3. Outdoor Advertising Campaign

Advertisements (Supplementary Figure 3) were placed at bus stops, on pharmacy bags, on digital screens (“Amscreens”) in GP practices, and in local press. The advertisements were developed using insights from a range of sources, including the Department of Health's Healthy Foundations Segmentation Model [25], previous campaigns, and input from the target audience. The creative design featured people chosen to reflect local demographic characteristics and emphasised the effectiveness of screening with the headline “this little kit saves lives from bowel cancer” and byline “the test can detect invisible early signs of bowel cancer” plus an additional banner highlighting ease of use “it's easier than you think.” The cost of placing the advertisements was £150,000 in total, although costs vary depending on various factors, such as the time of year and location.

### 2.4. Measures

Anonymised data were provided by the BCSP for all men and women invited for bowel cancer screening in Greater London from 4th January 2013 to 5th April 2013 and 4th January 2014 to 5th April 2014. Data included the date that each recipient was invited, the date they were subsequently sent a gFOBT kit, the date that participants initially returned their first kit, the date they returned subsequent kits if required (e.g., if the original kit was spoilt or yielded a weak positive result), and the date that they were adequately screened (if at all). A recipient was defined as adequately screened if they reached a definitive gFOBT outcome of either “normal” or “abnormal” within 12 weeks from the date they were sent an invitation.

An area-level measure of socioeconomic deprivation of the invitee (Index of Multiple Deprivation (IMD) score [26]) was included as a continuous variable, where a low IMD score (e.g., 10) denoted a lower level of deprivation and a high score (e.g., 60) denoted a higher level of deprivation. Previous screening status split all invitees into one of three categories: those who had not been invited to bowel screening before (“first-timers”), those who had been invited before and been adequately screened at least once (previously screened), and those who had been invited before and never been adequately screened (previous nonresponders). Gender and age at invitation were also included. Each invitee's designated intervention group was included (A: endorsement flyer, B: endorsement flyer and enhancement pack, C: endorsement flyer and advertising, and D: endorsement flyer, enhancement pack, and advertising).

### 2.5. Data Analysis

Analysis aimed to determine whether the interventions were associated with a significant increase in gFOBT uptake at 12 weeks after the invitation date, compared to controls, whilst controlling for other factors known to affect uptake. A secondary aim was to examine how the impact of the interventions varied across different demographic groups and to identify groups where the interventions appeared to have the greatest impact.

Multivariate logistic regression models were then used to model the probability of screening uptake in each intervention group compared to a predefined “control group,” whilst keeping all other variables at their mean values in the models. The control group included all those eligible for bowel cancer screening across London who were invited to complete a gFOBT from 4th January to 5th April 2013 or from 4th January to 5th April 2014 and who did not receive an intervention (and also were not excluded under any of the criteria below). Models controlled for other variables that were available and known to affect uptake, including gender, age, previous screening status, and deprivation. In order to control for any underlying trend in daily uptake across both four-month periods studied and over the two years, the date that each invitee was sent their gFOBT kit was also included as a discrete noncategorical variable.

The logistic regression models were then used to explore differences in the size of each intervention's impact on uptake in different invitee groups (i.e., in different previous screening status groups, at various deprivation levels, and in males and females). We compared the difference in predicted uptake probabilities between each intervention group and controls in one invitee group (e.g., first-timers) to the difference in predicted uptake probabilities between each intervention group and controls in another group (e.g., previous nonresponders).

### 2.6. Data Validation and Exclusions

Eighteen invitees were excluded because their screening records contained inconsistent data. An additional 11,795 invitees were excluded from analysis because the invitee's age at invitation was under 60 or over 74 years, they were missing an IMD score, or they were sent an invitation but not later sent a kit (i.e., the date the kit was sent was missing). The reasons for not being sent a kit include the invitee opting out of that screening episode out of choice or by notifying the London BCSP Hub of a significant medical condition (including cancer), the invitation being recorded as returned mail, the invitee's relocation outside the catchment area of the Hub, or their death. Therefore, uptake was calculated as the percentage of those sent kits that were adequately screened within 12 weeks, in order to better isolate the impact of the interventions. Uptake figures are therefore not directly comparable with figures published by BCSP.

### 2.7. Model Development and Sensitivity Analysis

Initial inspection of the data showed that different relationships between previous screening status, age, and uptake were apparent for 60–69-year-olds and 70–74-year-olds. Uptake amongst invitees included in the final analysis increases with age until 70 years (including a slight levelling-off between 66 and 69 years), after which uptake decreases. The percentage of invitees at each age that have been previously screened follows a similar pattern, increasing with age until 70 years and subsequently decreasing. This was most likely because the 70–74 age extension was not yet well established in all areas of London. Therefore, subsequent analyses were carried out separately for these two age groups, and the majority of the analysis focused on 60–69-year-olds, as we predicted that the relationships in the older age group would be likely to change as the age extension becomes better established in London.

Invitees from North East London sent their kits in 2014 could have been exposed to the advertising campaign, despite being in the control group or being in intervention group A or B. For example, an invitee in intervention group A in North East London who was sent their kit on 3rd February 2014 could have delayed returning their kit until after the advertising campaign began in North East London on 24th February, and then seen the advertising, and returned their kit in time to be adequately screened within 12 weeks of their invitation.

A sensitivity analysis was conducted to examine the effect of accounting for this contamination using different methods. A contamination variable was subsequently included in the final analysis, to adjust for the possible impact of “contaminated” records on uptake in the control group, intervention A, and intervention B. This method used the full sample available for each intervention group, whilst allowing the effect of contamination to vary between contaminated records from the control group, intervention group A, and intervention group B.

The final model included an interaction term between previous screening status and age, as these factors, and their effect on uptake, were already known to be closely related [13]. Previous models identified other interactions between predictor variables. The overall impact of the interventions on uptake was similar in these models, although in some versions of the model for 60–69-year-olds uptake amongst intervention group C was not significantly different from controls.

One model, which was not chosen as the final model, included an interaction between intervention group and previous screening status. This model showed that the impact of intervention B was smaller in first-timers (interaction term; OR = 0.82, *P* = 0.011) and previous nonresponders (interaction term; OR = 0.76, *P* = 0.001) than in previously screened invitees, whilst the impact of D was also smaller in first-timers (interaction term; OR = 0.80, *P* = 0.016) than in previously screened invitees (data not shown). These results are not consistent with the final model, and therefore results in this paper suggesting that the impact was greater in first-timers should be viewed with some caution.

## 3. Results

### 3.1. Impact of Interventions

#### 3.1.1. Greater London

Screening uptake amongst intervention group A (the CRUK endorsement flyer alone) was not associated with an increase in gFOBT uptake at 12 weeks compared to controls, in either age group (60–69 years: 43.0% versus 43.4%, OR = 0.99, *P* = 0.68; 70–74 years: 45.1% versus 46.7%, OR = 0.94, *P* = 0.381) (Tables 2, 3, and 4). Modelled uptake amongst intervention group B (the CRUK endorsement flyer plus kit enhancement pack) was significantly higher (45.1%) than controls (43.4%) (OR = 1.07, *P* = 0.047), amongst 60–69-year-olds, but there was no difference observed in 70–74-year-olds (OR = 1.03, *P* = 0.739).

#### 3.1.2. North East London

Modelled uptake amongst intervention group C (the CRUK endorsement flyer plus advertising) was significantly higher (45.6%) than controls (43.4%) in 60–69-year-olds (OR = 1.09, *P* = 0.027), but not in 70–74-year-olds (OR = 0.88, *P* = 0.318).

Intervention group D (the CRUK endorsement flyer plus the kit enhancement pack and advertising) was associated with the largest increases in modelled uptake compared to controls, in both age groups (60–69 years: 49.5% versus 43.4%, OR = 1.28, *P* < 0.001; 70–74 years: 53.9% versus 46.7%, OR = 1.34, *P* = 0.001). Intervention group D was the only intervention in 70–74-year-olds with significantly higher uptake compared to controls.

### 3.2. Covariates

Multivariate analysis accounted for the effects of other variables known to be associated with uptake (Tables 2 and 3). In 60–69-year-olds, uptake was substantially lower amongst people who were being invited to screening for the first time (“first-timers,” OR = 0.17, *P* < 0.001) compared to those who had been successfully screened at least once before (“previously screened”). Odds of uptake were lower still amongst those who had been invited before but had not yet been screened successfully (“previous nonresponders,” OR = 0.05, *P* < 0.001), compared to previously screened invitees (Table 2). The model showed that the relationship between age at invitation and uptake was different in first-timers (OR = 0.94, *P* < 0.001) and previous nonresponders (OR = 0.91, *P* < 0.001), compared with previously screened invitees. Consistent with previous studies, there were decreasing odds of uptake with increasing deprivation (IMD score, OR = 0.98, *P* < 0.001). These patterns were also present in 70–74-year-olds (Table 3).

In 60–69-year-olds, odds of uptake were significantly higher amongst “contaminated” invitees in the control group (OR = 1.09, *P* = 0.006), intervention A (OR = 1.26, *P* = 0.001), and intervention B (OR = 1.29, *P* = 0.001) who could have been exposed to advertising, compared to uncontaminated invitees (Table 2). Although contamination was not a significant predictor of uptake in 70–74-year-olds, the variable was also included in this model to account for this design limitation in a consistent way.

The date that each invitee was sent their gFOBT kit was included in the model as a discrete noncategorical variable, to control for any underlying trend in uptake across both four-month periods studied and over the two years. This showed that there was also a decrease in the odds of uptake each day amongst both 60–69-year-olds (OR = 0.9997, *P* < 0.001) and 70–74-year-olds (OR = 0.9996, *P* < 0.001).

### 3.3. Variation in Impact of Interventions by Invitee Characteristics

Further analysis showed that the impact of the intervention groups on gFOBT uptake varied depending on the invitees' previous screening status. For example, keeping other characteristics (i.e., age, deprivation, gender, and date the kit was sent) constant, in 60–69-year-old first-timers, modelled uptake was 39.4% in intervention group D and 33.7% in controls (Table 4). In 60–69-year-olds who were previously screened, uptake was 83.0% in intervention group D and 79.3% in controls, whilst in previous nonresponders it was 15.4% and 12.5%, respectively. This equates to a larger (5.7%) absolute increase in uptake between intervention group D and controls within first-timers, compared to previously screened invitees (3.8%) and previous nonresponders (3.0%). Similar patterns were identified in 70–74-year-olds (Table 4).

The impact of the interventions also varied with the deprivation level of the invitee.  Table 5 shows modelled uptake for selected IMD score points, from 10 to 60 (the majority of invitees included in the model had IMD scores that fell within this range). Keeping other characteristics constant, in less deprived 60–69-year-olds (i.e., those who had an IMD score of 10), modelled uptake was 54.8% in intervention group D, compared to 48.6% in controls (Table 5). In contrast, in more deprived 60–69-year-olds (i.e., IMD score of 60), uptake was 35.7% in intervention group D, compared to 30.2% in controls. This equates to a larger (6.2%) absolute increase in uptake between intervention group D and controls amongst less deprived invitees, compared to the increase amongst more deprived invitees (5.5%). Similar patterns were identified in 70–74-year-olds (Table 5).

In 60–69-year-olds, there was little variation in the impact of the interventions between males and females. Gender was not included in the final analysis of 70–74-year-olds, as it was not a significant predictor of uptake in this age group.

## 4. Discussion

Results indicate that a combination of mailed interventions together (CRUK endorsement flyer and kit enhancement pack) was associated with an increase in uptake that was small but significantly higher than for controls, among adults invited for bowel cancer screening across London. In North East London, the combination of these mailed items together with advertising in the local area had a substantial impact. The size of the impact varied considerably by previous screening status and deprivation.

In Greater London, the CRUK endorsement flyer alone (intervention group A) did not significantly increase uptake. This contrasts with findings by Hewitson et al., which showed that an endorsement from a GP in the form of a separate letter sent with gFOBT kits was associated with a 6 percentage point increase in uptake compared with controls [15]. Both the CRUK flyer in this study and the materials in the study by Hewitson et al. were sent together with NHS gFOBT kits, suggesting that although Cancer Research UK is a well known charity, it could be that a letter from someone's own GP provides a stronger endorsement. Further research could investigate whether endorsement from an organisation such as Cancer Research UK has more impact if sent separately from the gFOBT kits.

Adding the kit enhancement pack to the endorsement flyer (intervention group B) was associated with a significant, if small, increase in uptake compared to controls in 60–69-year-olds (45.1% versus 43.4%). As the flyer alone did not affect uptake, this suggests that the enhancement pack may have helped reduce barriers to completing the gFOBT kits. This contrasts with a previous study in the Netherlands that found that a faeces collection paper sent with Faecal Immunochemical Test (FIT) kits was not associated with significantly increased participation in bowel cancer screening [22]. However, the kit enhancement packs in this pilot were sent as a separate mailing (two days after the gFOBT kits were sent), so it is possible that this effect was a result of the additional communication acting as a reminder. Future research could further explore the individual impact of the enhancement pack on uptake and relative impact of the gloves or “poo catchers.”

In North East London, the CRUK endorsement flyer and advertising campaign were associated with significantly higher uptake (45.6%) compared to controls (43.4%) when trialled together (intervention group C). To our knowledge, this is the first study to assess the impact of an advertising campaign that aimed to improve uptake of gFOBT in the UK, although findings support those of a previous study that identified an association between media activity around the publication of the UK Flexible Sigmoidoscopy Trial and increased early uptake of gFOBT [24]. It would be interesting to see the impact of an advertising campaign in isolation and extend the follow-up period to assess longevity.

The addition of the kit enhancement pack to the CRUK endorsement flyer and advertising (intervention group D) was associated with the largest increases in uptake compared to controls, in both age groups (60–69 years: 49.5% versus 43.4%; 70–74 years: 53.9% versus 46.7%). This suggests that the kit enhancement packs had an additive effect, possibly by further helping to reduce barriers to completing screening and/or appealing to a different set of invitees. This is in line with evidence suggesting that multichannel interventions tend to be more effective than those using just one approach [23].

The impact of the interventions varied depending on previous screening status, with intervention group D having the largest impact in first-timers amongst both 60–69-year-olds and 70–74-year-olds. This is encouraging, because previous studies have shown that completing screening once is a strong predictor of future participation, with 86.6% of those who complete screening once, participating in the next round [13]. These results should be viewed with some caution, as an alternative model that included an interaction between intervention group and previous screening status suggested that the impact of intervention groups B and D was smaller in first-timers.

The increase in uptake in intervention group D amongst previous nonresponders, whilst smaller in absolute terms (3.0% for 60–69-year-olds), is also noteworthy given the substantially lower underlying uptake in this group (the relative increase is 23.7%). A cohort study in the NHS BCSP Southern Hub by Lo et al. [13] confirmed that a majority (66.2%) of people invited to gFOBT screening will eventually accept the offer at least once, suggesting that repeated attempts to engage with previous nonresponders are important.

Less encouraging, however, was the finding that the interventions increased uptake by a smaller amount amongst more deprived invitees. This is disappointing, given the well-documented social gradient in uptake, and means that if rolled out, care needs to be taken to ensure that interventions do not exacerbate existing inequalities [7]. As nonresponders to screening and more deprived populations are generally less responsive to follow-up, future research could potentially use surveys and focus groups of the eligible population to further explore reasons why these interventions may appeal more or less to different demographic groups [18].

### 4.1. Strengths and Limitations

A strength of this study lies in the large dataset of individual level records included in the final analysis (*n* = 205, 541), which made it possible to detect absolute differences in uptake in the magnitude of 1 to 2% and also take into account other demographic characteristics that could have confounded the effects of the intervention groups on uptake. There is considerable variation in screening uptake from week to week and year on year. The analysis included a number of covariates known to be associated with this variation (i.e., deprivation, gender, and previous screening status), as well as the date the invitee was sent their kit, in order to control for this variation.

However, it is possible that the impact of the interventions on gFOBT uptake was not statistically significant amongst 70–74-year-olds because of the smaller sample sizes in this model and a subsequent lack of power. The number of records included in the intervention groups in this model ranged from 1,609 for intervention A to 466 for intervention group C (Table 1).

Another strength comes from a relatively low proportion of cases excluded due to missing or inconsistent data (*n* = 11, 813 or 5.4% of the original data), offering a relatively complete snapshot of uptake for the eligible population. Furthermore, interventions were trialled within the BCSP, enhancing external validity of the study.

However, some limitations of this study should be noted. As previously mentioned, it was not possible to compare the impact of interventions run across London with those run only in North East London, due to population differences that we could not account for in analysis. Some factors known to affect uptake could not be controlled for (e.g., ethnicity and marital status), because they are not routinely collected by the Bowel Cancer Screening Programme. Therefore, comparisons between interventions carried out within London (interventions A and B) and within North East London (interventions C and D) should be interpreted with caution.

As previously mentioned, some invitees from North East London in the control group and intervention groups A and B could potentially have been exposed to the advertising campaign before completing their kits. However, models included a contamination variable to account for this.

It is also not known what proportion of first-timers and previous nonresponders could have moved into the catchment area of the BCSP London Hub from another country of the UK or abroad and previously been invited (or potentially successfully screened) at their previous residence.

To our knowledge, this was the only pilot running in the BCSP London Hub area at the time, but it is possible that there were other activities, taking place outside the Hub, which also aimed to improve uptake and could have had an impact on screening uptake and our results.

## 5. Conclusions

The greatest increase in gFOBT uptake at 12 weeks was seen in North East London, where all three interventions (CRUK endorsement flyer, kit enhancement pack, and advertising campaign) were combined. The CRUK endorsement letter together with the advertising campaign was also associated with a small but significant increase in uptake compared to controls in North East London. In Greater London, the CRUK endorsement letter, together with the kit enhancement pack, also had a small positive impact on uptake. However, the CRUK endorsement letter alone did not affect uptake across London.

These findings suggest that a combination of interventions designed to improve awareness and understanding of the English NHS Bowel Cancer Screening Programme, along with packs that ease the stool sampling process, are associated with increased gFOBT uptake. Future research should investigate the effectiveness of each intervention component, such as which part of the kit enhancement packs (gloves or “poo catchers”) is most effective in increasing gFOBT uptake, and attempt to identify the mechanisms through which they have an effect. However, adoption of the Faecal Immunochemical Test (FIT) by the BCSP may also address some of the practical barriers to gFOBT screening.

## Supplementary Material

Supplementary material contains images of the piloted interventions, including a copy of the CRUK endorsement flyer included with gFOBT kit mailings, a picture of the gFOBT kit enhancement pack, and an example of a poster used in the outdoor advertising campaign.

## Figures and Tables

**Table 1:** Total sample of invitees in each intervention group and number included in the final multivariate logistic regression models.

Intervention group	Dates invited (control group)/dates kits sent (interventions)	Total sample of invitees aged 60–74 years (before exclusions)	Number of invitees aged 60–69 years (after exclusions)	Number of invitees aged 70–74 years (after exclusions)
Control group	4th January–5th April 2013, 4th January–5th April 2014 (excluding trialled interventions)	187,554	145,427	31,959

Greater London interventions				
(A) CRUK endorsement flyer only	27th January–4th February 2014	10,286	8,093	1,609
(B) CRUK endorsement flyer, plus kit enhancement pack	5th February–11th February 2014	9,096	7,148	1,475

North East London interventions				
(C) CRUK endorsement flyer, plus advertising campaign	24th February–13th March 2014	5,121	4,332	466
(D) CRUK endorsement flyer, plus kit enhancement pack and advertising campaign	24th March–14th April 2014	5,297	3,980	1,052

Total	—	217,354	168,980	36,561

**Table 2:** Multivariate logistic regression model for gFOBT uptake amongst 60–69-year-olds (*n* = 168,980).

Adequately screened at 12 weeks	Odds ratio	*P* value	95% lower confidence limit	95% upper confidence limit
Intervention group (reference = control group)				
A	0.99	0.680	0.92	1.05
B	1.07	0.047	1.00	1.15
C	1.09	0.027	1.01	1.18
D	1.28	<0.001	1.18	1.39
Contamination variable (reference = uncontaminated)				
Contaminated-control	1.09	0.006	1.03	1.17
Contaminated-A	1.26	0.001	1.10	1.44
Contaminated-B	1.29	0.001	1.11	1.51
Age at invitation (reference = 60 years)	1.004	0.353	1.00	1.01
Previous screening status (reference = previously screened)				
First-timers	0.17	<0.001	0.16	0.18
Previous nonresponders	0.05	<0.001	0.05	0.06
Interaction term: previous screening status × age at invitation				
First-timers	0.94	<0.001	0.92	0.96
Previous nonresponders	0.91	<0.001	0.90	0.93
IMD score	0.98	<0.001	0.98	0.99
Gender (reference 0 = female, 1 = male)	0.83	<0.001	0.81	0.85
Date the kit was sent (days)	0.9997	<0.001	1.00	1.00
Constant	5653.54	<0.001	1369.18	23344.31

**Table 3:** Multivariate logistic regression model for gFOBT uptake amongst 70–74-year-olds (*n* = 36,561).

Adequately screened at 12 weeks	Odds ratio	*P* value	95% lower confidence limit	95% upper confidence limit
Intervention group (reference = control group)				
A	0.94	0.381	0.81	1.08
B	1.03	0.739	0.89	1.19
C	0.88	0.318	0.69	1.13
D	1.34	0.001	1.13	1.59
Contamination variable (reference = uncontaminated)				
Contaminated-control	1.03	0.806	0.84	1.26
Contaminated-A	1.08	0.725	0.72	1.61
Contaminated-B	1.03	0.906	0.64	1.67
Age at invitation (reference = 70 years)	0.96	0.001	0.94	0.98
Previous screening status (reference = previously screened)				
First-timers	0.09	<0.001	0.07	0.12
Previous nonresponders	0.02	<0.001	0.02	0.02
Interaction term: previous screening status × age at invitation				
First-timers	1.12	0.018	1.02	1.24
Previous nonresponders	1.24	<0.001	1.19	1.29
IMD score	0.99	<0.001	0.98	0.99
Date the kit was sent (days)	0.9996	<0.001	1.00	1.00
Constant	11733.30	<0.001	484.95	283884.90

**Table 4:** Modelled absolute and relative differences^i^ in gFOBT uptake across the intervention groups^ii^ overall and within different previous screening status groups.

	(1) Analysis of 60–69-year-olds						(2) Analysis of 70–74-year-olds				
Previous screening status	Control group (*n* = 145,427)	(A) CRUK endorsement flyer only (*n* = 8,093)	(B) CRUK endorsement flyer, plus kit enhancement pack (*n* = 7,148)	(C) CRUK endorsement flyer, plus advertising campaign (*n* = 4,332)	(D) CRUK endorsement flyer, plus kit enhancement pack and advertising campaign (*n* = 3,980)		Control group (*n* = 31,959)	(A) CRUK endorsement flyer only (*n* = 1,609)	(B) CRUK endorsement flyer, plus kit enhancement pack (*n* = 1,475)	(C) CRUK endorsement flyer, plus advertising campaign (*n* = 466)	(D) CRUK endorsement flyer, plus kit enhancement pack and advertising campaign (*n* = 1,052)
Predicted probability of uptake in each intervention group and control group											
Overall	43.4%	43.0%	45.1%	45.6%	49.5%		46.7%	45.1%	47.3%	43.6%	53.9%
Previously screened	79.3%	79.0%	80.4%	80.7%	83.0%		78.5%	77.4%	78.9%	76.4%	83.0%
First-timers	33.7%	33.4%	35.3%	35.7%	39.4%		29.9%	28.6%	30.4%	27.4%	36.3%
Previous nonresponders	12.5%	12.3%	13.3%	13.5%	15.4%		11.1%	10.5%	11.3%	9.9%	14.3%

Absolute difference in modelled uptake in each significant intervention group, compared to control group											
Overall	43.4%	—	+1.7%	+2.2%	+6.1%		46.7%	—	—	—	+7.3%
Previously screened	79.3%	—	+1.1%	+1.4%	+3.8%		78.5%	—	—	—	+4.5%
First-timers	33.7%	—	+1.6%	+2.0%	+5.7%		29.9%	—	—	—	+6.4%
Previous nonresponders	12.5%	—	+0.8%	+1.0%	+3.0%		11.1%	—	—	—	+3.2%

Relative difference in modelled uptake in each significant intervention group, compared to control group											
Overall	43.4%	—	+4.0%	+5.0%	+14.2%		46.7%	—	—	—	+15.6%
Previously screened	79.3%	—	+1.4%	+1.8%	+4.8%		78.5%	—	—	—	+5.7%
First-timers	33.7%	—	+4.7%	+5.9%	+17.0%		29.9%	—	—	—	+21.5%
Previous nonresponders	12.5%	—	+6.3%	+7.9%	+23.7%		11.1%	—	—	—	+28.9%

Data shows the predicted probability of uptake using multivariate logistic regression models, after accounting for other factors known to affect uptake.

Data shown for separate analyses of (1) 60–69-year-olds and (2) 70–74-year-olds.

^i^Absolute and relative differences may not exactly equal differences in modelled uptake due to rounding.

^ii^Differences are not shown for intervention groups that were not statistically significant overall.

**Table 5:** Modelled absolute and relative differences^i^ in gFOBT uptake across the intervention groups^ii^, at selected IMD scores (levels of deprivation).

	(1) Analysis of 60–69-year-olds						(2) Analysis of 70–74-year-olds				
IMD score (deprivation level)	Control group (*n* = 145,427)	(A) CRUK endorsement flyer only (*n* = 8,093)	(B) CRUK endorsement flyer, plus kit enhancement pack (*n* = 7,148)	(C) CRUK endorsement flyer, plus advertising campaign (*n* = 4,332)	(D) CRUK endorsement flyer, plus kit enhancement pack and advertising campaign (*n* = 3,980)		Control group (*n* = 31,959)	(A) CRUK endorsement flyer only (*n* = 1,609)	(B) CRUK endorsement flyer, plus kit enhancement pack (*n* = 1,475)	(C) CRUK endorsement flyer, plus advertising campaign (*n* = 466)	(D) CRUK endorsement flyer, plus kit enhancement pack and advertising campaign (*n* = 1,052)
Predicted probability of uptake in each intervention group and control group											
10—lower deprivation	48.6%	48.3%	50.3%	50.8%	54.8%		51.1%	49.5%	51.7%	48.0%	58.3%
20	44.7%	44.4%	46.4%	46.9%	50.9%		47.4%	45.8%	48.0%	44.3%	54.6%
30	40.9%	40.6%	42.6%	43.0%	47.0%		43.7%	42.1%	44.3%	40.7%	50.9%
40	37.2%	36.9%	38.8%	39.3%	43.1%		40.1%	38.6%	40.7%	37.2%	47.2%
50	33.6%	33.3%	35.2%	35.6%	39.3%		36.6%	35.1%	37.2%	33.8%	43.6%
60—higher deprivation	30.2%	29.9%	31.7%	32.1%	35.7%		33.2%	31.8%	33.8%	30.6%	40.0%

Absolute difference in modelled uptake in each significant intervention group, compared to control group											
10—lower deprivation	48.6%	—	+1.7%	+2.2%	+6.2%		51.1%	—	—	—	+7.2%
20	44.7%	—	+1.7%	+2.2%	+6.2%		47.4%	—	—	—	+7.3%
30	40.9%	—	+1.7%	+2.1%	+6.1%		43.7%	—	—	—	+7.2%
40	37.2%	—	+1.6%	+2.1%	+5.9%		40.1%	—	—	—	+7.1%
50	33.6%	—	+1.6%	+2.0%	+5.7%		36.6%	—	—	—	+7.0%
60—higher deprivation	30.2%	—	+1.5%	+1.9%	+5.5%		33.2%	—	—	—	+6.7%

Relative difference in modelled uptake in each significant intervention group, compared to control group											
10—lower deprivation	48.6%	—	+3.6%	+4.5%	+12.7%		51.1%	—	—	—	+0.7%
20	44.7%	—	+3.9%	+4.9%	+13.8%		47.4%	—	—	—	+0.4%
30	40.9%	—	+4.1%	+5.2%	+14.9%		43.7%	—	—	—	+0.2%
40	37.2%	—	+4.4%	+5.6%	+16.0%		40.1%	—	—	—	+0.2%
50	33.6%	—	+4.7%	+5.9%	+17.0%		36.6%	—	—	—	+0.1%
60—higher deprivation	30.2%	—	+4.9%	+6.2%	+18.0%		33.2%	—	—	—	+0.1%

Data shows the predicted probability of uptake using multivariate logistic regression models, after accounting for other factors known to affect uptake.

Data shown for separate analyses of (1) 60–69-year-olds and (2) 70–74-year-olds.

^i^Absolute and relative differences may not exactly equal differences in modelled uptake due to rounding.

^ii^Differences are not shown for intervention groups that were not statistically significant overall.
