# Supplementary material for: Piloting the Impact of Three Interventions on Guaiac Faecal Occult Blood Test Uptake within the NHS Bowel Cancer Screening Programme
Source: Biomed Res Int. 2015 Oct 7;2015:928251. doi: 10.1155/2015/928251 (PMC4615211; doi:10.1155/2015/928251)

Supplementary Material

Refer to supplementary material for a copy of the CRUK endorsement flyer, a picture of the gFOBT enhancement pack, and an example of a poster used in the outdoor advertising campaign.

Supplementary Figure 1: CRUK endorsement flyer

CANCER RESEARCH UK

Message from Martin Ledwick,
Head Cancer Information Nurse, Cancer Research UK

Bowel cancer screening saves lives – in fact, it's predicted that it will save over 2,000 lives a year by 2025.

A simple and private test
Across London, every month over 17,000 men and women complete and return their NHS bowel cancer screening kit. Like them, you can do the test in the privacy of your own home. Most people say it is easy to do.

Detects invisible signs
Bowel cancer screening is meant for people who have no symptoms at all. It looks for tiny traces of blood in the poo that can't be seen by the naked eye. It can detect bowel cancer at an early stage, when with effective treatment more people survive.

You have a choice whether or not to do bowel cancer screening, so please read the 'Bowel Cancer Screening - The Facts' booklet, which you were sent with your screening invitation, to help you decide.

We recommend people complete the NHS bowel cancer screening kit.

If you have any questions call the NHS Bowel Cancer Screening Helpline
0800 707 6060
(Mon-Fri 9.00am – 5.00pm)

Supplementary Figure 2: gFOBT enhancement pack

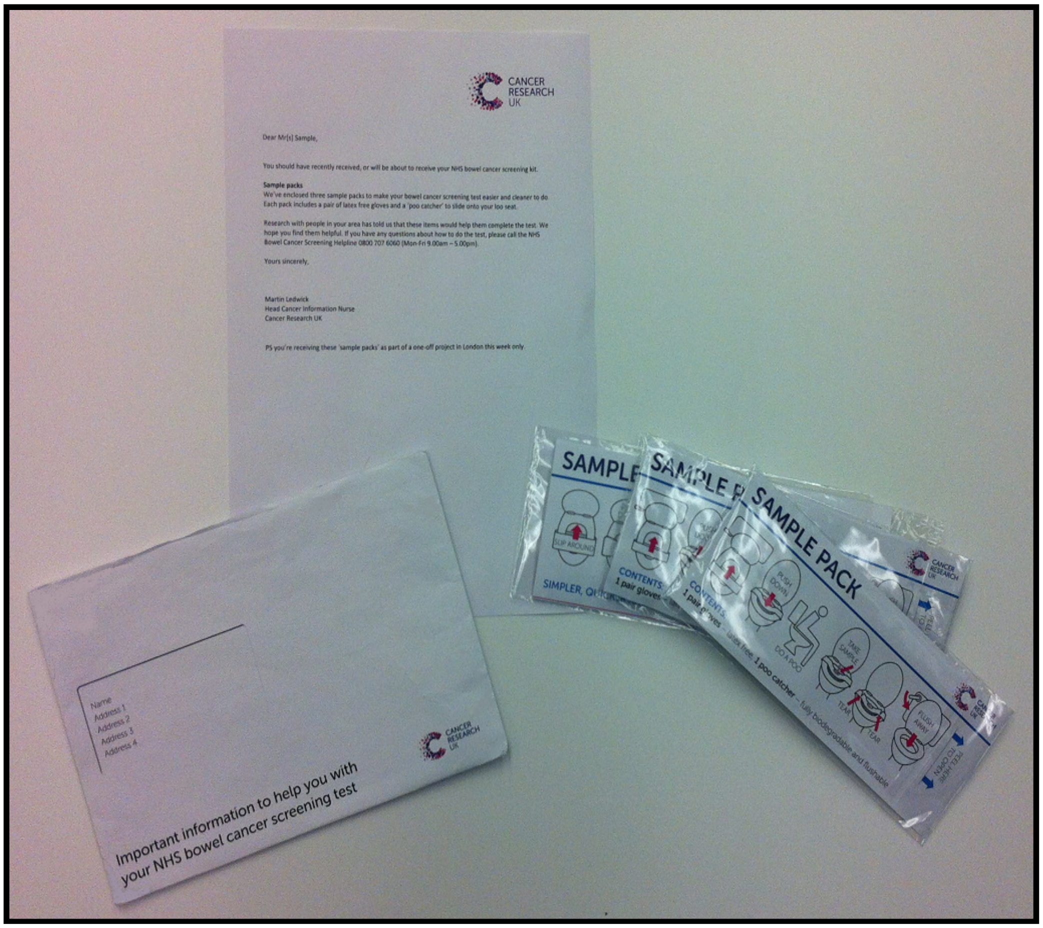

Supplementary Figure 3: Example of poster used in the outdoor advertising campaign

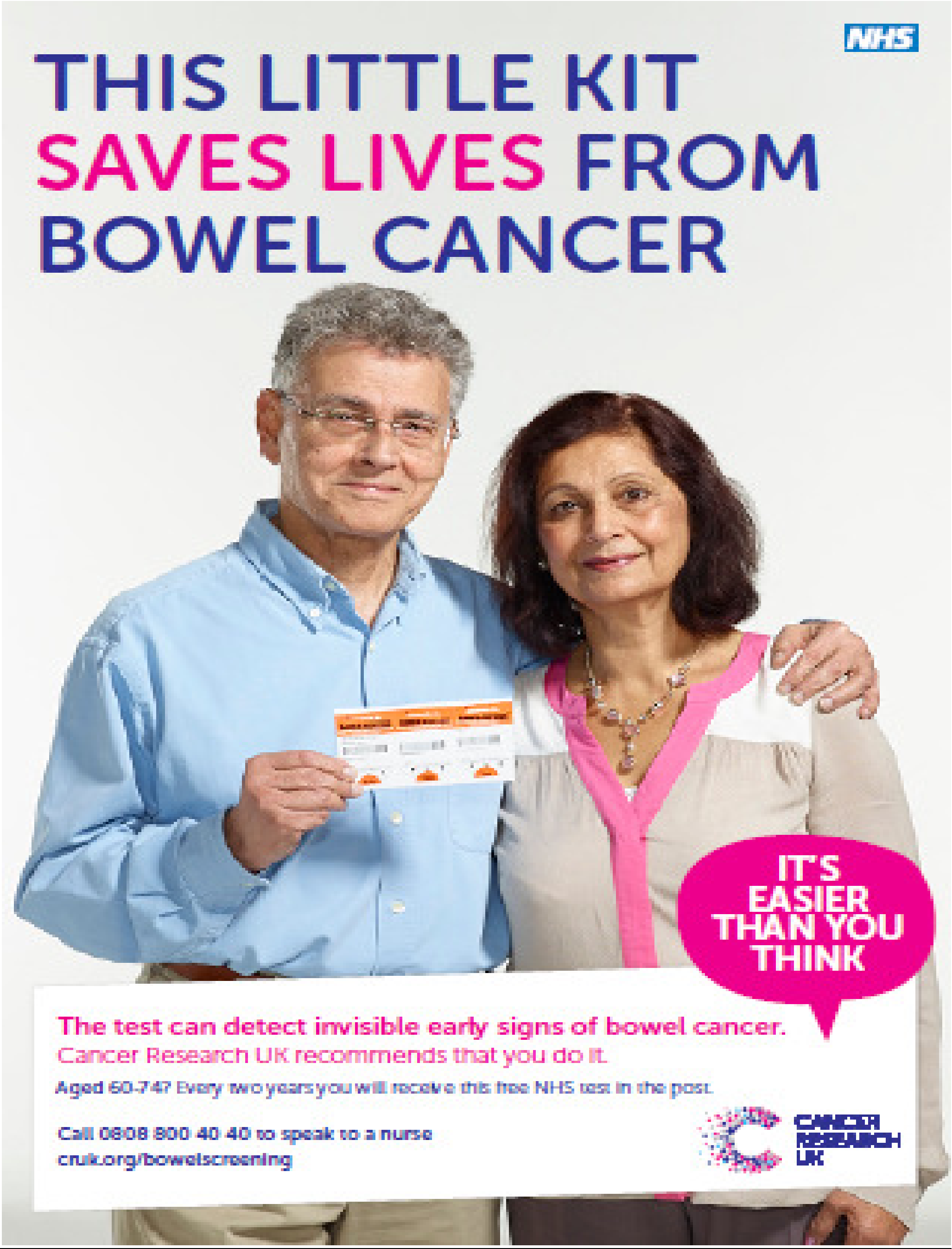

Supplement: Supplementary file 1 — Supplementary material contains images of the piloted interventions, including a copy of the CRUK endorsement flyer included with gFOBT kit mailings, a picture of the gFOBT kit enhancement pack, and an example of a poster used in the outdoor advertising campaign. [file 928251.f1.pdf]
